# Extensive Metabolite Profiling in the Unexploited Organs of Black Tiger for Their Potential Valorization in the Pharmaceutical Industry

**DOI:** 10.3390/life11060544

**Published:** 2021-06-10

**Authors:** Jianfei Gao, Kangning Xiong, Wei Zhou, Weijie Li

**Affiliations:** 1 Institute of Mountain Resources, Guizhou Academy of Sciences, Guiyang 550001, China; nf1985@yeah.net (J.G.); weili_55@163.com (W.L.); 2 State Engineering Technology Institute for Karst Desertification Control of China, School of Karst Science, Guizhou Normal University, Guiyang 550001, China; 3 Guizhou Industry Polytechnic College, Guiyang 550008, China; zhouwei_460@163.com

**Keywords:** black tiger, metabolite profile, polyphenol, nutraceutical, plant valorization, medicinal plant

## Abstract

Black tiger (*Kadsura coccinea* (Lem.)) has been reported to hold enormous pharmaceutical potential. The fruit and rhizome of black tiger are highly exploited in the pharmaceutical and other industries. However, the most important organs from the plant such as the leaf and stem are considered biowastes mainly because a comprehensive metabolite profile has not been reported in these organs. Knowledge of the metabolic landscape of the unexploited black tiger organs could help identify and isolate important compounds with pharmaceutical and nutritional values for a better valorization of the species. In this study, we used a widely targeted metabolomics approach to profile the metabolomes of the *K. coccinea* leaf (KL) and stem (KS) and compared them with the root (KR). We identified 642, 650 and 619 diverse metabolites in KL, KS and KR, respectively. A total of 555 metabolites were mutually detected among the three organs, indicating that the leaf and stem organs may also hold potential for medicinal, nutritional and industrial applications. Most of the differentially accumulated metabolites between organs were enriched in flavone and flavonol biosynthesis, phenylpropanoid biosynthesis, arginine and proline metabolism, arginine biosynthesis, tyrosine metabolism and 2-oxocarboxylic acid metabolism pathways. In addition, several important organ-specific metabolites were detected in *K. coccinea*. In conclusion, we provide extensive metabolic information to stimulate black tiger leaf and stem valorization in human healthcare and food.

## 1. Introduction

Black tiger (*Kadsura coccinea* (Lem.)) is a perennial evergreen climbing woody vine belonging to the family *Schisandraceae*. It has ovate-elliptic-shaped leaves, solitary with hexagonally structured skin developed by each carpel, one–three flat unisexual flowers and large globose fruits with seeds in the mericarp [1]. The color, shape and size of fruits and leaves vary among *Kadsura* species. *K. coccinea* is widely distributed in Guizhou, Guangxi, Yunnan and southern China, consumed by the local population as fresh fruit and used as a key ingredient in local juice and wine [2]. Its rhizome has high medicinal value and is used in folk medicine to treat gastric and duodenal ulcers, acute gastroenteritis, rheumatoid arthritis, bruises, swelling and pain, dysmenorrhea and many other conditions [3,4]. Modern medicinal research showed that the black tiger rhizome has anti-tumor, anti-HIV, anti-inflammatory, hepatoprotective and antioxidant effects, attracting widespread attention in the field of phytomedicine [5,6,7]. It has been demonstrated that the leaf and stems can relieve rheumatic pain in the bones, chronic enteritis, acute gastritis [8,9] and immunologic hepatic fibrosis [10,11]. The stem has also been used to prevent and treat rheumatic and arthritic diseases, with anti-nociceptive and anti-inflammatory effects [12,13,14].

Research on the chemical composition and biological activities of black tiger has mainly been focused on the rhizome, which has lignans, triterpenes, sesquiterpenes, steroids and amino acids as the main chemical components [2,6,7]. At present, more than 70 types of lignan compounds such as schizandrin, isovaleroyl-binankadsurin and more than 80 triterpenoid compounds such as coccinone A and coccinone B acids have been isolated from the black tiger rhizome [2,4,6,7]. However, the chemical composition of other organs of black tiger including the leaf and stem has not been reported [2,4,6,8,9,10,14]. The leaf and stem of black tiger plants are usually discarded after harvest and represent waste biomass. Determining the metabolome profiles of black tiger leaves and stems could facilitate the identification of the common and unique metabolites in these organs for potential valorization.

Metabolomics is a functional “omics” technique for performing qualitative and quantitative studies on plant metabolites and other relevant constituents [15,16,17]. It has been widely utilized to study plant metabolism and food quality. Common metabolites such as alkaloids, flavonoids, glycosides, organic acids, saponins and steroids have been studied and engineered for improving pharmaceutical and phytochemical values of medicinal plants [18,19]. To harness the medicinal, nutritional and nutraceutical values of black tiger, it is important to identify the functional compounds and understand their biological activities in the different organs.

In this study, we performed comparative metabolome profiling on the leaves, stems and roots of *K. coccinea*. Unique and differentially accumulated metabolites involved in important biosynthetic pathways were identified and characterized among the three organs. This is the first report of a widely targeted metabolome profiling of black tiger which may serve as a valuable resource for further functional study on the species.

## 2. Materials and Methods

### 2.1. Study Area, Plant Material, Growth and Sampling

The study was conducted on leaves, stems and roots of black tiger plants (*Kadsura coccinea* (Lem.)), locally named “*Hei Laohu*” and cultivated outdoor in 2017 in Yunyan District, Guiyang City, Guizhou Province, China (latitude 106°42′04″ and longitude 26°34′48″). Four-year-old black tiger plants were used in the experiment. Seedlings were transplanted from the nursery to resin plastic pots containing understory humus and yellow soil (*v*/*v* = 1:1). The branches were pruned from time to time, and the plants were watered regularly. We selected three plants with similar growth patterns for sampling. Plants were harvested with a shovel to ensure they were neatly pulled from the soil. We cut 3 main roots and fine roots from each plant as root samples. We also sampled 3 main vines about 10 cm above the ground surface as stems and harvested 6 fresh leaves. All samples were taken in triplicate and transported in 10 mL cryotubes. These tubes were subsequently labeled as KL1–3 (leaves), KR1–3 (roots) and KS1–3 (stems) and quickly placed in liquid nitrogen. The samples were stored on ice and transported to the laboratory and then stored in a −80 °C ultra-low temperature refrigerator.

### 2.2. Sample Preparation and Extraction

Biological samples were freeze dried by a vacuum freeze dryer (Scientz-100F, Scientzbio, Ningbo, China) and crushed using a mixer mill (MM 400, Retsch, Haan, Germany) with a zirconia bead for 1.5 min at 30 Hz. An amount of 100 mg of lyophilized powder was mixed in 1.2 mL 70% methanol solution, vortexed for 30 s every 30 min 6 times and placed in a refrigerator at 4 °C overnight. Following centrifugation at 12,000 rpm for 10 min, the extracts were filtrated (SCAA-104, 0.22 μm pore size; ANPEL, Shanghai, China; http://www.anpel.com.cn/, accessed on 12 June 2020) before UPLC-MS/MS analysis.

### 2.3. Organ-Specific Metabolome Profiling and Analyses

Widely targeted metabolome analyses were performed by the Wuhan MetWare Biotechnology Co., Ltd. (Wuhan, China) on the leaf, root and stem samples. Each organ sample consisted of three biological replicates with a total of nine samples. Sample preparation and analysis, metabolite detection and computations were undertaken by the Wuhan MetWare Biotechnology Co., Ltd. (Wuhan, China), following their standard procedures as described by Wang et al. [20].

### 2.4. Metabolite Determination

The sample extracts were analyzed using a UPLC-ESI-MS/MS system (UPLC, SHIMADZU Nexera X2, www.shimadzu.com.cn/, accessed on 8 June 2021; MS, Applied Biosystems 4500 Q TRAP, www.appliedbiosystems.com.cn/ accessed on 8 June 2021). The analytical conditions were as follows: UPLC: column, Agilent SB-C18 (1.8 µm, 2.1 × 100 mm). The mobile phase consisted of solvent A, pure water with 0.1% formic acid, and solvent B, acetonitrile with 0.1% formic acid. Sample measurements were performed with a gradient program that employed the starting conditions of 95% A, 5% B. Within 9 min, a linear gradient to 5% A, 95% B was programmed, and a composition of 5% A, 95% B was kept for 1 min. Subsequently, a composition of 95% A, 5.0% B was adjusted within 1.10 min and kept for 2.9 min. The flow velocity was set as 0.35 mL per min. The column oven was set to 40 °C, and the injection volume was 4 μL. The effluent was connected to an ESI-triple quadrupole-linear ion trap (QTRAP)-MS [21]. The analytical conditions were adapted from Chen et al. [17]. Metabolite quantification was conducted using multiple-reaction monitoring (MRM) [22] and the self-built MetWare database (MWDB) based on their standard metabolic operating procedures [15,17]. 

### 2.5. KEGG Annotation and Enrichment Analysis

Identified metabolites were annotated using the Kyoto Encyclopedia of Genes and Genomes (KEGG) compound database [23] (http://www.kegg.jp/kegg/compound/, accessed on 8 June 2021). Annotated metabolites were then mapped to the KEGG Pathway database (http://www.kegg.jp/30kegg/pathway.html accessed on 8 June 2021). Pathways with significantly regulated metabolites mapped were then fed into the Metabolite Set Enrichment Analysis (MSEA) database (https://www.metaboanalyst.ca/, accessed on 8 June 2021) [24], and their significance was determined as *p*-values of the hypergeometric tests.

### 2.6. Statistical Analyses

Quality control (QC) analysis was conducted to confirm the reliability of the data prior to the overall analyses. The QC sample was prepared by mixing sample extracts for insertion into every three samples to monitor the changes in repeated analyses. Data matrices with the intensity of the metabolite features from the nine samples were uploaded to the Analyst 1.6.1 software (AB Sciex, Redwood City, CA, USA) for statistical analyses. Partial least squares discriminant analysis (PLS-DA) was performed to maximize the metabolome differences between sample pairs. The relative importance of each metabolite to the PLS-DA model [25] was tested using the variable importance in projection (VIP) as a parameter. Metabolites with VIP ≥ 1 and fold change ≥ 2 or fold change ≤ 0.5 were considered as differential metabolites for group discrimination [25]. The hierarchical cluster analysis (HCA) results of samples and metabolites were presented as heatmaps using *pheatmap* in the R package (www.r-project.org, accessed on 8 June 2021) [26]. For HCA, normalized signal intensities of metabolites (unit variance scaling) were visualized as a color spectrum. Consequently, a metabolic pathway was constructed according to KEGG (http://www.genome.jp/kegg/, accessed on 8 June 2021) [23], and pathway analysis was performed using MetaboAnalyst (http://www.metaboanalyst.ca/, accessed on 8 June 2021) [27] based on the change in metabolite ion intensity compared with the corresponding controls.

## 3. Results

### 3.1. Widely Targeted Metabolome Profiling in Leaf, Stem and Root of Black Tiger

**Figure 1:**
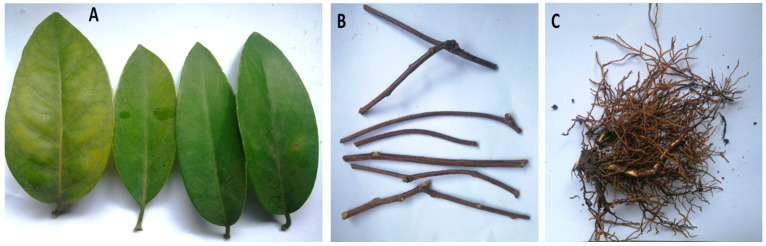
Organs of *Kadsura coccinea* used for metabolite profiling: (**A**) leaf, (**B**) stem and (**C**) root.

Fresh leaf, stem and root samples of four-year-old *K. coccinea* plants were collected and used for metabolite profiling (Figure 1A–C). The present study used UPLC-MS/MS to accurately profile the metabolites both qualitatively and quantitatively in the nine samples (three organs × three biological repeats). The leaves, stems and roots were designated as KL (KL1, KL2 and KL3), KS (KS1, KS2 and KS3) and KR (KR1, KR2 and KR3), respectively. The ion chromatograms of three different organs (KL, KS and KR) of *K. coccinea* samples are shown in Supplementary Figure S1.

**Figure 2:**
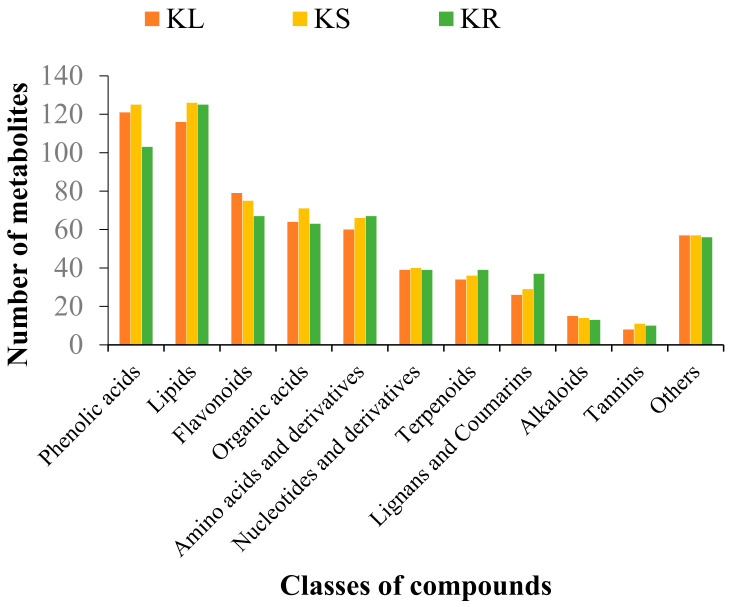
Number of metabolites detected in each class of compounds in different organs of *K. coccinea*, including leaf (KL), stem (KS) and root (KR).

We identified a total of 642, 650 and 619 metabolites in KL, KS and KR, respectively. The metabolites were clustered into 11 classes of compounds, namely, alkaloids, amino acids and derivatives, flavonoids, lignans and coumarins, lipids, nucleotides and derivatives, organic acids, phenolic acids, tannins, terpenoids and others (Figure 2). Among these, 555 metabolites were mutually detected among the three organs, while 55 (KL and KS), 11 (KL and KR) and 27 (KS and KR) metabolites were uniquely detected in the pairwise group of organs (Supplementary Figure S2; Supplementary Table S1). The high number of common compounds among the three organs indicates that the leaf and stem could be exploited for medicinal, nutritional and other industrial uses. 

**Figure 3:**
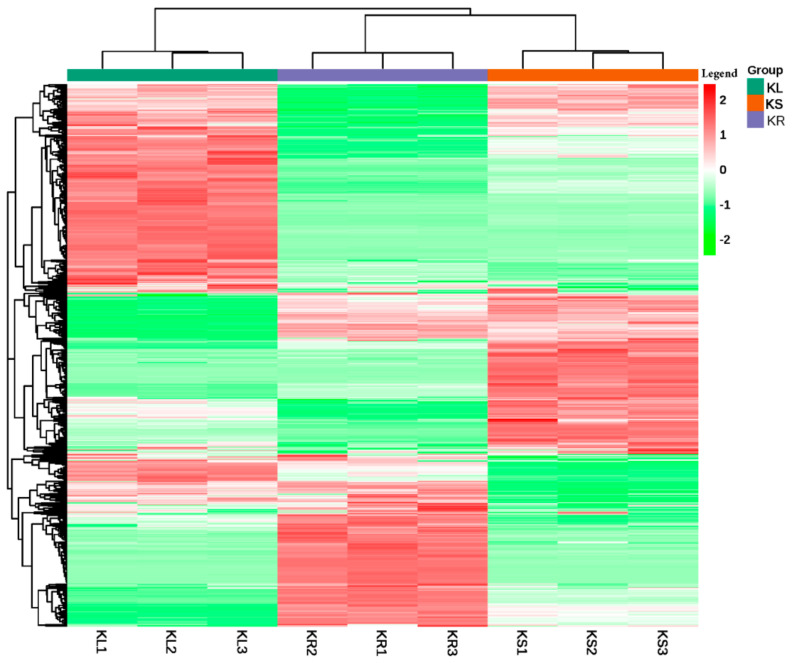
Hierarchical clustering based on the ion intensity of metabolites from leaf (KL), stem (KS) and root (KR) of *K. coccinea* represented by green, chocolate and purple colors at top of the figure. All analyses were conducted in triplicates, KL (KL1–KL3), KS (KS1–KS3) and KR (KR1–KR3). The color intensity of each metabolite is shown in the legend on right-hand side of the figure, and thus green color represents decreased abundance, red color represents increased abundance and white color represents not detected.

We performed cluster analysis on the samples based on the metabolite ion intensity. The three organs with their biological repeats were clustered into two main groups. Cluster I exclusively comprised KL samples, whereas Cluster II consisted of KS and KR samples (Figure 3). This suggests that KL is relatively different from either KS or KR. However, the metabolite concentration and composition in the stems and roots share relatively high similarity. 

### 3.2. Specific Metabolites in the Different Organs of K. coccinea 

Identification of unique metabolites among the three organs used in the present study will deepen our understanding of their potential uses in the food and pharmaceutical industries. We identified a number of specific metabolites in each organ. Leaf samples (KL) contained 21 unique metabolites with 7 phenolic acid compounds (5′-glucosyloxyjasmanic acid, dicaffeoylquinic acid-o-glucoside, 2-o-galloyl-glucose, chlorogenic acid methyl ester, 5-o-galloyl-methyl quinine ester, maleoyl-caffeoylquinic acid and p-hydroxyphenyl acetic acid) and 6 flavonoid compounds (kaempferol-3-o-arabinoside, quercetin-3-o-glucosyl(1→4)rhamnoside-7-o-rutinoside, avicularin (quercetin-3-o-α-l-arabinofuranoside), luteolin-7-o-glucoside (cynaroside), limocitrin-7-o-glucoside and kaempferol-3-o-neohesperidoside-7-o-glucoside) (Supplementary Table S1). Additionally, two compounds each from lignans, coumarins, organic acids and derivatives and one compound each from alkaloids, lipids, terpenoids and others were identified solely in the leaf sample (Supplementary Table S2a). 

Thirteen unique metabolites were found in the stem sample (KS), comprising seven phenolic acids, three organic acids and derivatives, two lipids and one other compound (Supplementary Table S2a). The seven phenolic acid compounds include o-anisic acid (2-methoxybenzoic acid), 4-hydroxy-3-methoxymandelate, 5-o-galloylshikimic acid, 1′-o-(3,4-dihydroxyphenethyl)-o-caffeoyl-glucoside, sinapyl alcohol, dimethyl phthalate and hydroquinone. 

Twenty-six metabolites were unique in the root sample (KR) (Supplementary Figure S1; Table 1). These consisted of 6 classes of compounds (i.e., 1 alkaloid, 3 flavonoids, 14 lignans and coumarins, 3 lipids, 1 phenolic acid and 4 terpenoids) (Table 1). These unique compounds may be the basis for the wide usage of black tiger roots. 

### 3.3. Identification of Differentially Accumulated Metabolites in Different Organs of Black Tiger 

**Figure 4:**
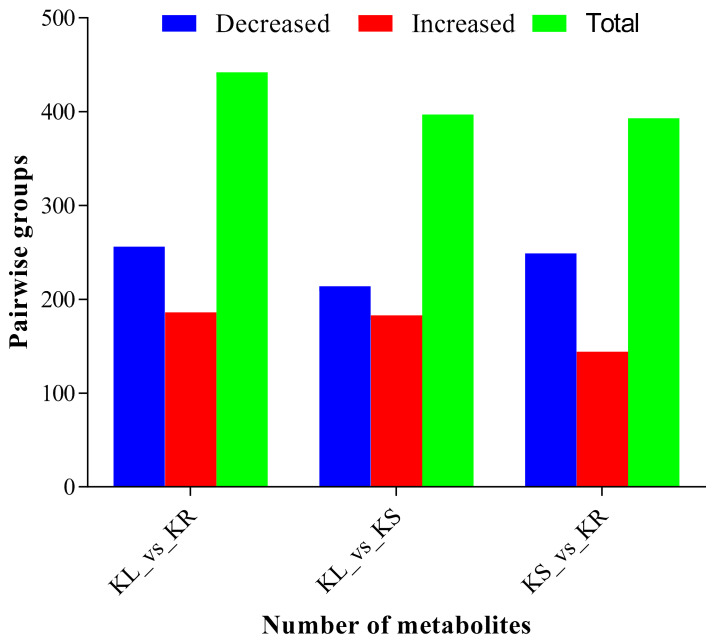
Differentially accumulated metabolites (DAMs) in leaf (KL), stem (KS) and root (KR) of *K. coccinea* in pairwise comparisons. “Decreased” and “increased” represent number of DAMs that decreased and increased in abundance, respectively, while “total” is the summation of number of DAMs that decreased and increased in abundance. Partial least squares discriminant analysis thresholds of log2fold change ≥ 1 and variable importance in projection ≥ 1 were used to screen for DAMs.

Upon application of PLS-DA with thresholds of log2FC ≥ 1 and VIP ≥ 1 (Supplementary Figure S3), the highest number (442) of differentially accumulated metabolites (DAMs) was found in KL_vs_KR, out of which 256 decreased in abundance (−), while 186 increased in abundance (+) in the KL_vs_KR group (Figure 3). This was followed by KL_vs_KS with 397 DAMs (−214 and +183) and KS_vs_KR with 393 DAMs (−249 and +144) (Figure 4). The high proportion of DAMs detected between the different organs further highlights their specific metabolome composition. These DAMs were dominated by phenolic acids, lipids and flavonoids, whilst tannins were the least identified. The DAMs were further divided into six sub-clusters based on their accumulation patterns in the different organs (Supplementary Figure S4). Metabolites in each of these six sub-clusters were highly enriched in specific organs. For instance, metabolites of sub-clusters 1, 5 and 6 were highly enriched in KR, while those of sub-clusters 2 and 3 were enriched in KL and KS (Supplementary Figure S4).

**Figure 5:**
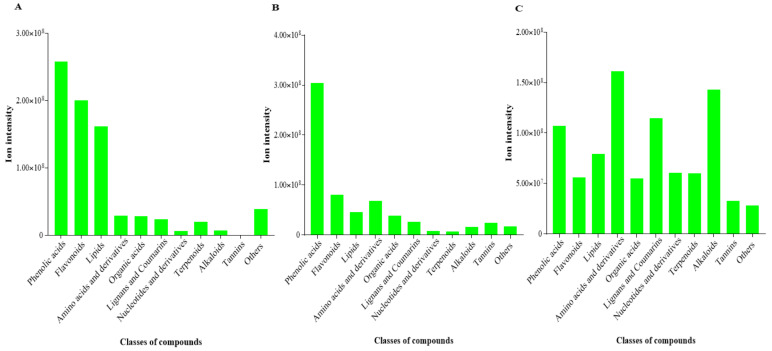
Abundance of compound class of differentially accumulated metabolites detected in different organs of *K. coccinea*. (**A**) Leaf (KL). (**B**) Stem (KS). (**C**) Root (KR). Each bar represents summation of ion intensities of compound classes.

The most abundant class of compounds (based on summation of ion intensities) detected in KL was phenolic compounds followed by flavonoid compounds (Figure 5). Similarly, the two top classes of compounds in KS were phenolic acids and flavonoids (Figure 5). Conversely, the amino acids and derivatives and alkaloids were the most abundant metabolites in KR (Figure 5). 

**Figure 6:**
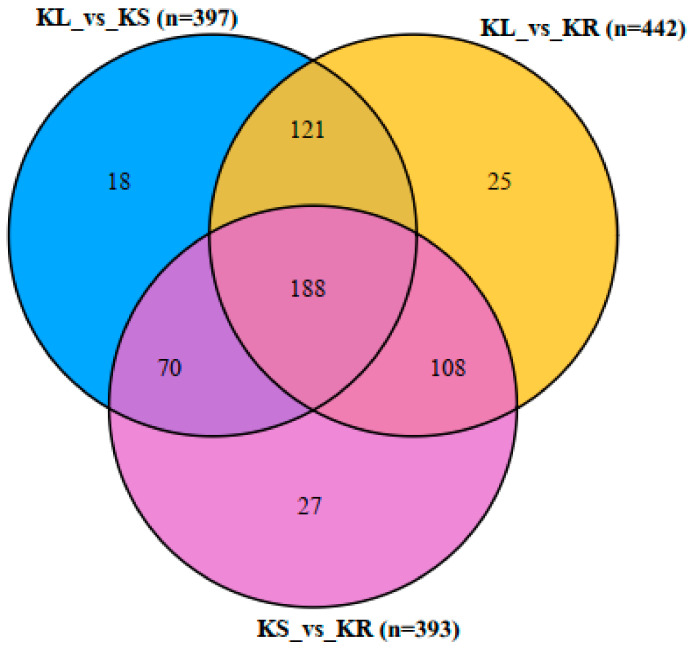
Venn diagram of differentially accumulated metabolites (DAMs) among the three pairwise groups (KL_vs_KR; KL_vs_KS; and KS_vs_KR). Leaf, stem and root of *K. coccinea* are designated as KL, KS and KR, respectively. n represents the number of DAMs detected in each pairwise group. Partial least squares discriminant analysis thresholds of log2 fold change ≥1 and variable importance in projection ≥ 1 were used to screen for DAMs.

Comparatively, 188 DAMs (33% of the total DAMs) were detected mutually among the three pairwise groups (KL_vs_KR, KL_vs_KS and KS_vs_KR), while 70–121 DAMs were detected in two of the pairwise groups (Figure 6; Supplementary Table S5). However, KL_vs_KS, KL_vs_KR and KS_vs_KR had 18, 25 and 27 unique DAMs. This indicates that several metabolites are present in the three organs concurrently at different levels of abundance. This supports our earlier assertion that the leaf and stem of *K. coccinea* may be useful for food, pharmaceutical and industrial uses. 

### 3.4. Pathway Enrichment Analyses of Differentially Accumulated Metabolites among the Three Organs of K. coccinea

**Figure 7:**
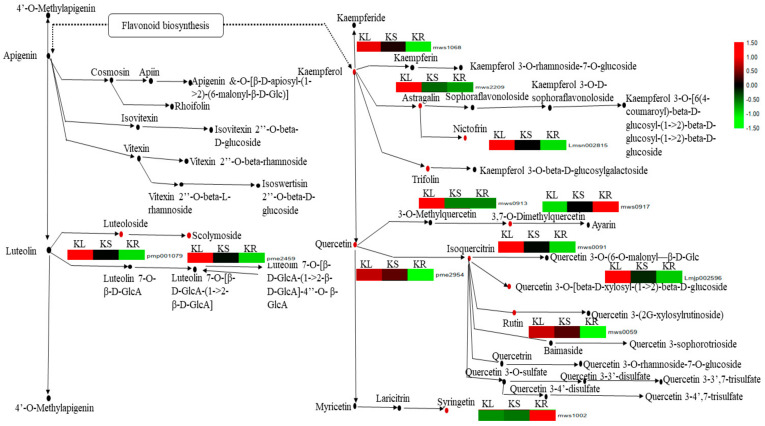
Metabolites involved in flavone and flavonol biosynthesis pathway. Metabolites with black dot denote non-differentially accumulated metabolites, while those with red dot denote differentially accumulated metabolites detected in *K. coccinea* leaf (KL), stem (KS) and root (KR). The heatmap near each differentially accumulated metabolite gives the level of abundance in each organ. Those with red and green colors indicate increase and decrease in abundance, while black indicates abundance near zero (see legend on right side of the figure). The abundance level of each metabolite was transformed by log_10_.

We subjected the DAMs detected in the pairwise groups to KEGG pathway enrichment analyses, yielding several significantly enriched pathways (*p*-value < 0.05). The most enriched pathway among the three pairwise groups was flavone and flavonol biosynthesis (Table 2; Supplementary Figure S4A–C). This prominent pathway had 12 DAMs with varied levels of abundance in KL, KS and KR (Figure 7). Out of these, 10 DAMs (luteoloside, scolymoside, kaempferol, astragalin, nictofrin, trifolin, quercetin, isoquercitrin, quercetin 3-o-[beta-d-xylosyl-(1→2)-beta-d-glucoside and rutin) showed significantly higher abundance in KL than either in KS or KR (Figure 7). The remaining two DAMs (3,7-o-dimethylquercetin and syringetin) were more abundant in KR compared to KL and KS. These results show that the *K. coccinea* leaf organ (and, to a lesser extent, the stem) is highly endowed with flavone- and flavonol-related metabolites.

Phenolic acids are produced in plants through the phenylpropanoid biosynthesis pathway with shikimic acid [28,29]. Sixteen phenolic compounds were only accumulated in KS and KR (Table 3). Of these, caffeic aldehyde, sinapyl alcohol, 5-o-caffeoylshikimic acid and syringin increased in abundance in KS compared to KR (Table 3). 

Another enriched pathway detected was arginine and proline metabolism, with 16 DAMs (Table 2 and Table 4). These comprised 2, 11 and 3 metabolites from alkaloids, amino acids and derivatives and organic acid compounds, respectively (Table 4). Among these, two alkaloid compounds (p-coumaroylputrescine and n-feruloylagmatine), three amino acids and derivates (trans-4-hydroxy-L-proline, s-adenosyl-l-methionine and guanidinoacetate) and two organic acids (4-acetamidobutyric acid and γ-aminobutyric acid) were abundant in both KL and KR but absent in KS (Table 4). Conversely, five amino acids and derivatives (L-aspartic acid, n-acetyl-l-glutamic acid, L-glutamine, L-proline and n-α-acetyl-l-ornithine) and one organic acid (α-ketoglutaric acid) were not identified in KR but mostly exhibited high abundance in KS and KL (Table 4). The remaining three amino acids and derivatives (L-ornithine, L-arginine and L-glutamic acid) were abundantly accumulated in the three organs (Table 4).

Phenylalanine and, to a lesser extent, tyrosine are the two main amino acids that are usually involved in the synthesis of phenolic acids in plants [30]. We detected tyrosine metabolism as one of the enriched pathways with 11 DAMs (3 amino acids and derivatives, and 8 phenolic acids) (Table 2 and Table 5). These compounds were highly enriched only in KL and KS (Table 5). 

In addition to the above enriched pathways, the 2-oxocarboxylic acid metabolism pathway was also enriched and included 19 DAMs (10 amino acids and derivatives, and 9 organic acids) (Table 2 and Table 6). These DAMs were abundant exclusively in KL and KS organs (Table 6). 

The above pathway enrichment analyses revealed that the three organs (KL, KS and KR) share a number of metabolites in common, while many metabolites are unique or significantly abundant in a single organ. 

## 4. Discussion

Metabolome profiling has become a state-of-the-art technique for practical genomic research due to the current advancement in mass spectrometric innovations [31,32]. Nearly 200,000 compounds have been profiled in plants, including approximately 10,000 secondary metabolites [33,34]. In this study, widely targeted metabolome profiling was adopted to explore the metabolites available in the leaves, stems and roots of *K. coccinea*. The study aimed at unraveling the potential metabolites in the leaf or stem which may play similar or unique functions to those found in the root, which is the main exploited organ [5,6,35,36]. The black tiger root holds significant economic, medicinal and nutritional value. However, the present study reveals that the stem and root share a large number of common metabolites as evidenced from our hierarchical clustering analysis (Figure 3). Thus, the stem may be as useful as the root. Notwithstanding, the high number of metabolites (555) mutually detected among the leaf, stem and root underscores the potential utility of the leaf and stem, in addition to the root (Supplementary Figure S1; Supplementary Table S1). For example, the leaf and stem contain high levels of quercetin and its derivatives (Supplementary Table S3), which account for 50% of the total dietary flavonoids in fruits, vegetables and beans and are essential in maintaining good human health as a result of their ubiquitous antioxidant property [10]. Additionally, this compound has an antiviral activity against various viral strains along with other flavonoids [37]. It works as an anticancer agent by regulating the cell cycle in human breast cancer MCF-7 cells [38]. Moreover, Tanwar and Modgil [39] reported several pharmacological effects of quercetin in various diseases such as neurogenerative disorders, inflammation, liver disorders, cardiovascular diseases and bacterial and fungal infections.

Each organ was found to have unique compounds (Table 1; Supplementary Table S2a,b). For instance, the leaf contains kaempferol-3-o-neohesperidoside-7-o-glucoside (flavonoid), which has recently been isolated and characterized to have antimicrobial [40], antioxidant [41], anticancer [42], neuroprotective [43], antidiabetic [44], immunomodulatory [45], anti-osteoporotic and antiestrogenic [46], anxiolytic [47], analgesic [48] and anti-allergic activities [45]. These varied properties of kaempferol-3-o-neohesperidoside-7-o-glucoside in the leaf make it a potential candidate for pharmaceutical use. Chlorogenic acid methyl ester, a phenolic acid, was also exclusively detected in the leaf (Supplementary Table S2a), which is useful in the food, health, cosmetic and pharmaceutical industries due to its anti-mutagenic, anti-proliferative and antioxidant potentials [49,50]. 

Likewise, the stem contains 13 unique compounds made up of 2 lipids, 3 organic acids, 7 phenolic acids and other compounds (Supplementary Table S2b). These metabolites include hydroquinone which is readily oxidized into quinines and further undergoes ring opening to produce muconic, maleic and fumaric acid derivatives of the starting phenolic compounds [51]. Hydroquinone is mainly used as an inhibitor in polymerization to produce antioxidants from food, rubber, plastic and other industrial antioxidants [51]. It is also useful in the production of polyetheretherketone for use in advanced material engineering in the aerospace and automotive industries [52]. This further makes the stem of black tiger a potential candidate for commercial exploitation.

The root contains 26 unique metabolites of phenolic compounds (flavonoids, lignans, coumarins and phenolic acids), lipids, alkaloids and terpenoids (Table 1). The high abundance of unique phenolic compounds in the root may explain the high commercial demand on the root compared to the leaf and stem. Phenolic compounds play a vital role in defense responses and anti-aging, anti-inflammatory, antioxidant and anti-proliferative activities in humans [28,29]. Additionally, phenolic compounds, through oxidative stress management, can reduce the incidence and severity of diabetes, cancers and cardiovascular diseases [29]. The alkaloids (indole), lipids (LysoPE 15:0, LysoPE 18:0 and LysoPE 20:5) and terpenoids (kadcoccilactone A, madasiatic acid, 2α,3β,19α,23-tetrahydroxyurs-12-en-28-oic acid and kadcoccilactone M) (Table 1) are some of the most active constituents/secondary metabolites involved in wound healing [53], anti-inflammatory actions [54], anti-diabetic activity [55], anti-tumor action [56] and anxiolytic [57], antiviral [58] and anti-proliferative activities [59,60]. 

## 5. Conclusions

The present study employed a widely targeted metabolomics approach to profile the metabolites in the leaf, stem and root of *K. coccinea*. Diverse metabolites were identified and characterized. The different organs share large numbers of metabolites in common, indicating that either the leaf or stem could be exploited for its potential valorization. Our metabolomic profiling highlighted the unique metabolites housed in the leaves, stems and roots of black tiger, identifying them as candidates for valorization in diverse industries. The information provided here would be valuable for applications in plant biotechnology to enhance secondary metabolite production from *K. coccinea*. The unique metabolites identified in the leaves and stems call for further studies on their commercial exploitation. 

## Figures and Tables

**Table 1:** Unique metabolites detected in root of *K. coccinea*.

Index ^a^	Compounds	Class	Ion Intensity
pmb1096	Indole	Alkaloids	23,589.33
Lmjp003402	2′-Hydroxy-3,4,5,3′,4′,6′-hexameth-oxychalcone	Flavonoids	1277.59
Hmhp007382	Altisin	Flavonoids	1277.59
pmp000109	5,7,8,4′-Tetramethoxyflavone	Flavonoids	1277.59
Lmhn008558	Kadangustin I	Lignans and Coumarins	4380.50
pmp000934	Gomisin N	Lignans and Coumarins	4380.50
pmp000949	Benzoylisogomisin O	Lignans and Coumarins	4380.50
pmp000955	Schinsanlignone A	Lignans and Coumarins	4380.50
pmp000953	Gomisin J	Lignans and Coumarins	4380.50
pmp000950	Gomisin G	Lignans and Coumarins	4380.50
pmp000941	Iso-schisandrin ethyl alcohol	Lignans and Coumarins	4380.50
pmp000959	Schisantherin D	Lignans and Coumarins	4380.50
pmp000961	Angeloylgomisin Q	Lignans and Coumarins	4380.50
Lmhn009930	Kadangustin F	Lignans and Coumarins	4380.50
Lmhp010106	Acetylepigomisin R	Lignans and Coumarins	4380.50
mws2164	Schisantherin A	Lignans and Coumarins	4380.50
Lmhn008287	Kadsuralignan A	Lignans and Coumarins	4380.50
Lmhn010428	Kadsuralignan H	Lignans and Coumarins	4380.50
Lmhp008885	LysoPE 15:0	Lipids	1234.38
pmb0880	LysoPE 18:0	Lipids	1234.38
Lmhp008688	LysoPE 20:5	Lipids	1234.38
Lmtn002324	Benzyl-(2″-O-glucosyl) glucoside	Phenolic acids	1861.37
Xmhn007019	Kadcoccilactone A	Terpenoids	2582.27
Hmjn003948	Madasiatic acid	Terpenoids	2582.27
Lmsn009589	2α,3β,19α,23-Tetrahydroxyurs-12-en-28-oic acid	Terpenoids	2582.27
Xmhn007682	Kadcoccilactone M	Terpenoids	2582.27

^a^ Obtained from the self-built MetWare database.

**Table 2:** Enriched pathways of metabolites detected in leaf (KL), stem (KS) and root (KR) of *K. coccinea* by KEGG.

Enriched Pathways	KL_vs._KR		KL_vs._KS		KS_vs_KR	
	DAMs (%) ^a^	*p*-Value ^b^	DAMs (%) ^a^	*p*-Value ^b^	DAMs (%) ^a^	*p*-Value ^b^
Flavone and flavonol biosynthesis	12/164 (7.32)	< 0.01	11/157 (7.01)	0.01	11/135 (8.15)	< 0.01
Phenylpropanoid biosynthesis	-	-	-	-	16/135 (11.85)	< 0.01
Arginine and proline metabolism	11/164 (6.71)	0.02	-	-	-	-
Arginine biosynthesis	-	-	8/157 (5.10)	0.04	-	-
Tyrosine metabolism	-	-	11/157 (7.01)	0.03	-	-
2-Oxocarboxylic acid metabolism	-	-	19/157 (12.10)	0.03	-	-

^a^ represents differentially accumulated metabolites in relation to the total number of metabolites in the pathway; ^b^ indicates the significance level.

**Table 3:** DAMs involved in phenylpropanoid biosynthesis pathway in *K. coccinea* leaf (KL), stem (KS) and root (KR).

Index ^a^	Compounds	Class	KL	KS	KR
pmb0142	Caffeic aldehyde	Phenolic acids	-	27,205.00	-
mws0853	Sinapyl alcohol	Phenolic acids	-	12,342.67	202,186.67
Hmln002806	5-O-Caffeoylshikimic acid	Phenolic acids	-	74,540.67	35,183.33
mws0011	Syringin	Phenolic acids	-	98,131.33	20,744.33
pmb3074	5-O-p-Coumaroylquinic acid	Phenolic acids	-	343,880.00	121,613.33
mws0178	Chlorogenic acid	Phenolic acids	-	67,665.67	491,066.67
mws2212	Caffeic acid	Phenolic acids	-	69,809.00	-
pma0149	Sinapoyl malate	Phenolic acids	-	262,770.00	30,502.33
pmb0751	Trans-5-O-(p-Coumaroyl) shikimate	Phenolic acids	-	53,514.67	-
mws0009	Coniferaldehyde	Phenolic acids	-	98,313.33	17,062.00
HJN003	1-O-Sinapoyl-d-glucose	Phenolic acids	-	81,737.00	29,457.00
mws0093	Coniferyl alcohol	Phenolic acids	-	2,050,366.67	492,696.67
mws0898	Isoeugenol	Phenolic acids	-	62,786.67	19,059.67
mws0014	Ferulic acid	Phenolic acids	-	231,966.67	-
mws0906	Coniferin	Phenolic acids	-	364,470.00	42,258.00
Lmmn001643	2-Hydroxycinnamic acid	Phenolic acids	-	286,560.00	139,856.67

^a^ Obtained from on the self-built MetWare database.

**Table 4:** DAMs involved in arginine and proline metabolism in *K. coccinea* leaf (KL), stem (KS) and root (KR).

Index ^a^	Compounds	Class	KL	KS	KR
pmb0490	p-Coumaroylputrescine	Alkaloids	24,160.00	-	9.00
pmb0496	*N*-Feruloylagmatine	Alkaloids	383,776.67	-	33,663.33
mws0216	Trans-4-Hydroxy-l-proline	Amino acids and derivatives	7825.67	-	43,322.67
mws0219	l-Aspartic Acid	Amino acids and derivatives	524,910.00	2,464,966.67	-
mws0260	l-Arginine	Amino acids and derivatives	71,618.00	411,153.33	1,850,500.00
pme0006	l-Proline	Amino acids and derivatives	1,422,000.00	-	3,054,933.33
pme0014	l-Glutamic acid	Amino acids and derivatives	6,100,933.33	18,427,333.33	15,762,666.67
pme0066	Guanidinoacetate	Amino acids and derivatives	8755.87	-	2220.20
pme0075	*N*-Acetyl-l-glutamic acid	Amino acids and derivatives	351,523.33	115,753.33	-
pme0193	l-Glutamine	Amino acids and derivatives	284,776.67	1,102,136.67	-
pme2527	l-Ornithine	Amino acids and derivatives	2016.07	50,564.67	33,644.67
pme2735	S-Adenosyl-l-methionine	Amino acids and derivatives	64,168.67	-	16,608.00
Zmyn000155	*N*-α-Acetyl-l-ornithine	Amino acids and derivatives	431,730.00	1,393,533.33	-
pme0295	4-Acetamidobutyric acid	Organic acids	127,520.00	-	-
pme2380	α-Ketoglutaric acid	Organic acids	52,782.67	135,200.00	-
pme3011	γ-Aminobutyric acid	Organic acids	61,506.67	-	1,043,233.33

^a^ Obtained from the self-built MetWare database.

**Table 5:** DAMs involved in tyrosine metabolism in *K. coccinea* leaf (KL), stem (KS) and root (KR).

Index ^a^	Compounds	Class	KL	KS	KR
pme1002	l-Tyramine	Amino acids and derivatives	60,308.33	1,298,266.67	-
mws0250	l-Tyrosine	Amino acids and derivatives	1,057,733.33	442,476.67	-
pme3827	3,4-Dihydroxy-l-phenylalanine (L-Dopa)	Amino acids and derivatives	35,464.67	10,650.10	-
MA10014775	Hydroquinone	Phenolic acids	-	14,767.00	-
Lmbn001981	2,5-Dihydroxybenzaldehyde	Phenolic acids	125,690.00	894,363.33	-
mws2368	Tyrosol	Phenolic acids	4810.00	43,634.67	-
mws0182	p-Hydroxyphenyl acetic acid	Phenolic acids	25,320.00	-	-
mws0180	2,5-Dihydroxybenzoic acid; Gentisic Acid	Phenolic acids	936,700.00	12,454,333.33	-
pme1292	Homogentisic acid	Phenolic acids	128,503.33	16,948.00	-
pme2598	3,4-Dihydroxybenzeneacetic acid	Phenolic acids	-	55,338.33	-
pme0085	Rosmarinic acid	Phenolic acids	135,003.33	498,560.00	-

^a^ Obtained from the self-built MetWare database.

**Table 6:** DAMs involved in 2-oxocarboxylic acid metabolism in *K. coccinea* leaf (KL), stem (KS) and root (KR).

Index ^a^	Compounds	Class	KL	KS	KR
pme2527	l-Ornithine	Amino acids and derivatives	2016.07	50,564.67	-
mws0219	l-Aspartic Acid	Amino acids and derivatives	524,910.00	2,464,966.67	-
pme0026	l-Lysine	Amino acids and derivatives	4976.43	21,966.00	-
pme0014	l-Glutamic acid	Amino acids and derivatives	6,100,933.33	18,427,333.33	-
pme1210	l-Methionine	Amino acids and derivatives	16,588.67	141,423.33	-
pme0021	l-Phenylalanine	Amino acids and derivatives	3,238,400.00	975,633.33	-
Zmyn000155	*N*-α-Acetyl-l-ornithine	Amino acids and derivatives	431,730.00	1,393,533.33	-
mws0250	l-Tyrosine	Amino acids and derivatives	1,057,733.33	442,476.67	-
pme0075	*N*-Acetyl-l-glutamic acid	Amino acids and derivatives	351,523.33	115,753.33	-
mws0282	l-Tryptophan	Amino acids and derivatives	640,426.67	2,035,900.00	-
mws0823	3-Methyl-2-Oxobutanoic acid	Organic acids	14,985.00	40,875.00	-
mws0425	Citraconic acid	Organic acids	-	163,850.00	-
Lmbn001288	2-Hydroxy-2-methyl-3-oxobutanoic acid	Organic acids	70,115.67	29,645.67	-
pme2380	α-Ketoglutaric acid	Organic acids	52,782.67	135,200.00	-
Lmbn001609	2-Acetyl-2-Hydroxybutanoic Acid	Organic acids	13,660.67	32,683.67	-
Lmbn001676	3-Hydroxy-3-Methyl-2-Oxopentanoic Acid	Organic acids	13,364.67	-	-
mws0159	Phenylpyruvic acid	Organic acids	160,270.00	23,230.00	-
Lmbn001754	3-Isopropylmalic Acid	Organic acids	247,716.67	104,970.67	-
pmb3101	2-Isopropylmalic Acid	Organic acids	248,146.67	102,494.00	-

^a^ Obtained from the self-built Metware database.

## Data Availability

The datasets used and/or analyzed during the current study are available in the manuscript and its Supplementary Files.

## Supplementary Materials

The following are available online at https://www.mdpi.com/article/10.3390/life11060544/s1. Figure S1: UPLC-ESI-MS/MS system chromatograms of the three organs of *Kadsura coccinea* (Lem), Figure S2: Venn diagram of metabolites detected in each, Figure S3: Discriminant analysis of orthogonal partial least squares (OPLS-DA) of metabolites in three pairwise groups: (A) KL_vs_KS; (B) KL_vs_KR; (C) KS_vs_KR. KL, KS and KR represent the leaf, stem and root of *K. coccinea*, respectively, Figure S4: K-means clustering of differentially accumulated metabolites based on ion intensity among the three organs of *K. coccinea* (leaf (KL), stem (KS) and root (KR)). Figure S5: Pathway enrichment by KEGG of differentially accumulated metabolites (DAMs) detected among the three pairwise groups, Table S1: Ion intensities (untransformed) of metabolites mutually detected in at least two parts of Kadsura coccinea Lem (leaf (KL); stem (KS) and root (KR)), Table S2a: Twenty-one unique metabolites identified in the leaf of *K. coccinea*. Table S2b: Thirteen unique metabolites identified in the stem of *K. coccinea*. Table S3: One hundred and eighty-eight differentially accumulated metabolites mutually detected among the three pairwise groups (KL_vs_KR, KL_vs_KS and KS_vs_KR) in *K. coccinea*.

## Abbreviations

DAMs	Differentially accumulated metabolites
KL	Leaves of *K. coccinea*
KS	Stems of *K. coccinea*
KR	Roots of *K. coccinea*
KEGG	Kyoto Encyclopedia of Genes and Genomes
VIP	Variable Importance in Projection
