# Supplementary material for: Extensive Metabolite Profiling in the Unexploited Organs of Black Tiger for Their Potential Valorization in the Pharmaceutical Industry
Source: Life (Basel). 2021 Jun 10;11(6):544. doi: 10.3390/life11060544 (PMC8229443; doi:10.3390/life11060544)
Supplement: Supplementary file 1 [file life-11-00544-s001.zip › Supplementary Figure S1.pdf]

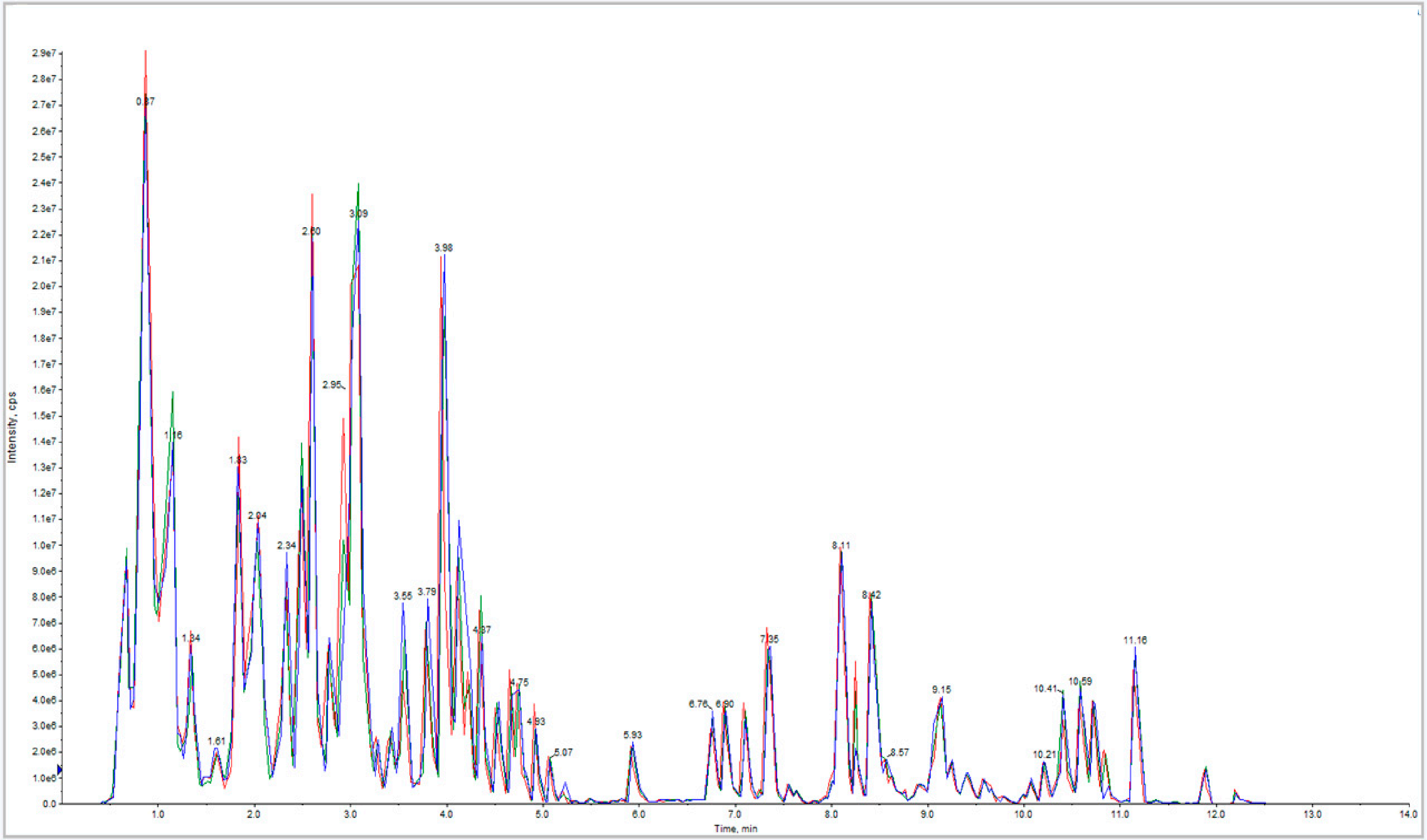

Supplementary Figure S1. UPLC-ESI-MS/MS system chromatograms of the three organs of *Kadsura coccinea* (Lem.) (Leaf, Stem and Root designated KL, KS and KR, respectively). The blue, red and green colors represent KL, KS and KR, respectively.
