# Supplementary material for: Extensive Metabolite Profiling in the Unexploited Organs of Black Tiger for Their Potential Valorization in the Pharmaceutical Industry
Source: Life (Basel). 2021 Jun 10;11(6):544. doi: 10.3390/life11060544 (PMC8229443; doi:10.3390/life11060544)
Supplement: Supplementary file 1 [file life-11-00544-s001.zip › Supplementary Figure S2.pdf]

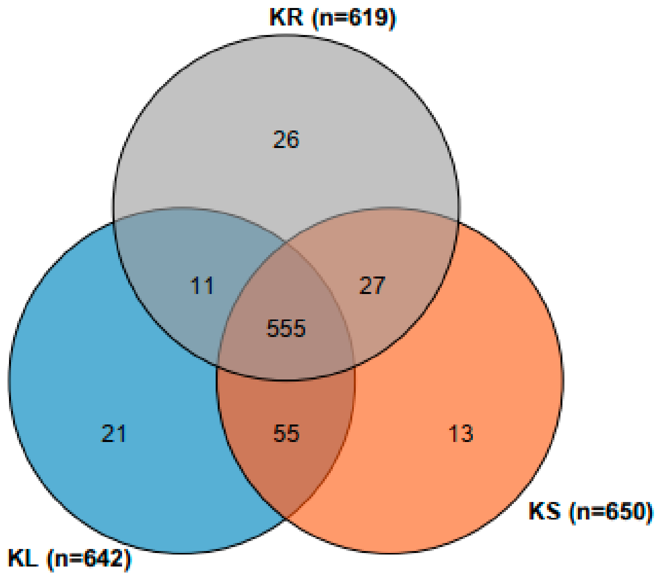

Supplementary Figure S2: Venn diagram of metabolites detected in each organ of *Kadsura coccinea* Lem: leaf, stem and root, represented by KL, KS and KR, respectively). Number of metabolites in each organ is denoted as n.
