# Supplementary material for: Extensive Metabolite Profiling in the Unexploited Organs of Black Tiger for Their Potential Valorization in the Pharmaceutical Industry
Source: Life (Basel). 2021 Jun 10;11(6):544. doi: 10.3390/life11060544 (PMC8229443; doi:10.3390/life11060544)
Supplement: Supplementary file 1 [file life-11-00544-s001.zip › Supplementary Figure S3.pdf]

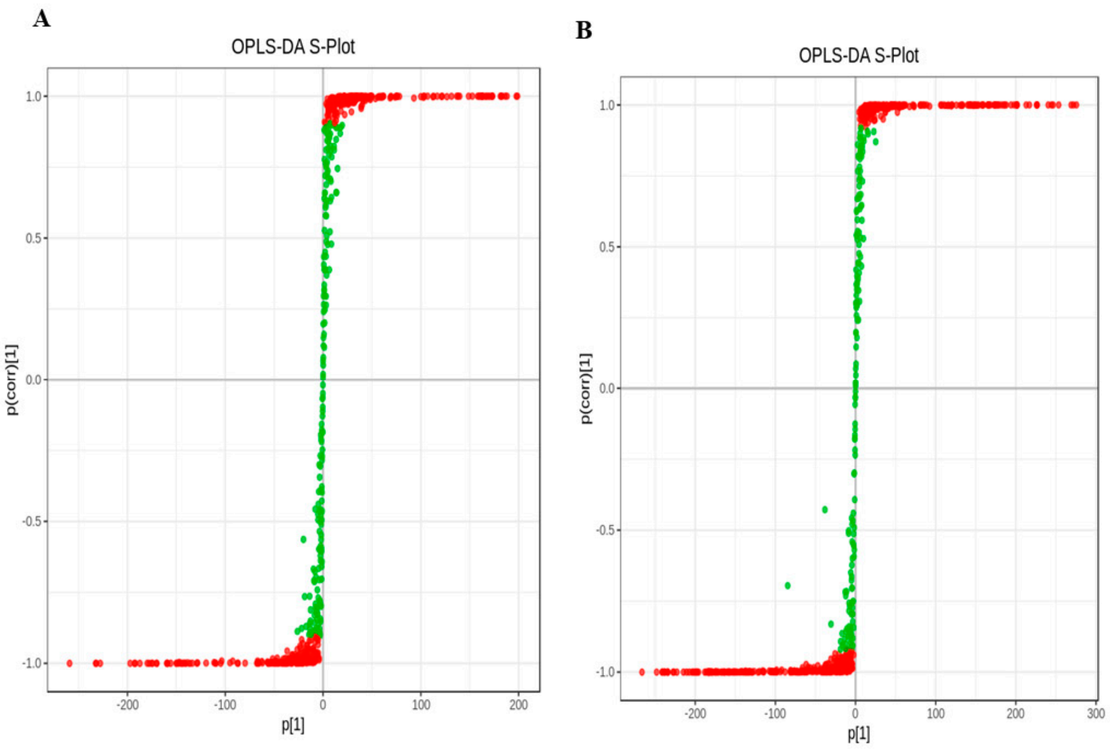

Supplementary Figure S3: Discriminant Analysis of Orthogonal Partial Least Squares (OPLS-DA) of metabolites in three pairwise groups. **(A)** KL_vs_KS, **(B)** KL_vs_KR, **(C)** KS_vs_KR. KL, KS and KR represent the leaf, stem and root of *Kadsura coccinea*, respectively. The abscissa represents the covariance between the principal component and the metabolite, and the ordinate represents the correlation coefficient between the principal component and the metabolite. Red dots represent significant metabolites while green dots are the non-significant metabolites.
