# Supplementary material for: Extensive Metabolite Profiling in the Unexploited Organs of Black Tiger for Their Potential Valorization in the Pharmaceutical Industry
Source: Life (Basel). 2021 Jun 10;11(6):544. doi: 10.3390/life11060544 (PMC8229443; doi:10.3390/life11060544)
Supplement: Supplementary file 1 [file life-11-00544-s001.zip › Supplementary Figure S5.pdf]

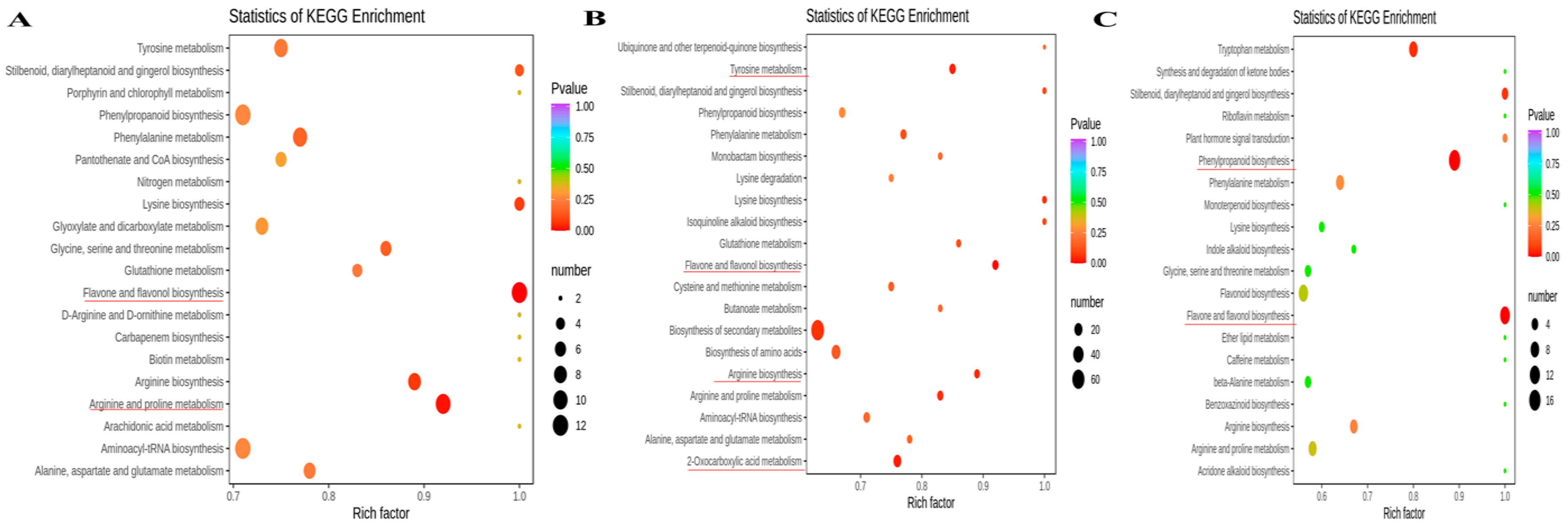

Supplementary Figure S5: Pathway enrichment by KEGG of differentially accumulated metabolites (DAMs) detected among the three pairwise groups. **(A)** KL_vs_KR. **(B)** KL_vs_KS. **(C)** KS_vs_KR. Pathways with p-values < 0.05 are underlined with red line. Organs used comprised leaf, stem and root of *Kadsura coccinea* Lem designated as KL, KS and KR, respectively.
