# Supplementary figures and images for: Extensive Metabolite Profiling in the Unexploited Organs of Black Tiger for Their Potential Valorization in the Pharmaceutical Industry
Source: Life (Basel). 2021 Jun 10;11(6):544. doi: 10.3390/life11060544 (PMC8229443; doi:10.3390/life11060544)

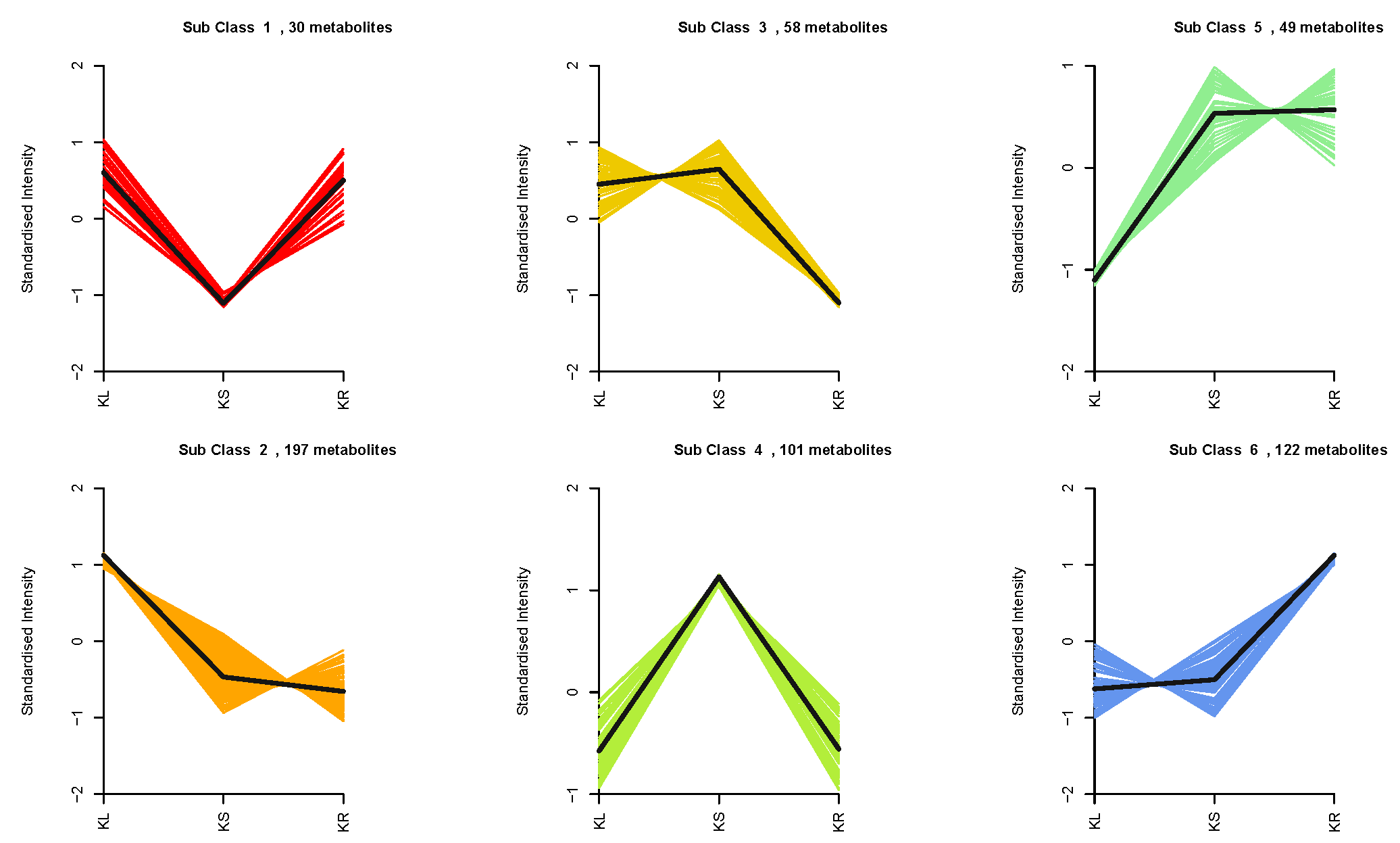

Supplement: Supplementary file 1 [file life-11-00544-s001.zip › Supplementary Figure S4.tiff]
